# A case of severe glutathione synthetase deficiency with novel GSS mutations

**DOI:** 10.1590/1414-431X20176853

**Published:** 2018-01-11

**Authors:** H. Xia, J. Ye, L. Wang, J. Zhu, Z. He

**Affiliations:** 1 Department of Neonatology, Xinhua Hospital, Shanghai Jiao Tong University School of Medicine, Shanghai, China; 2 Department of Pediatric Endocrinology and Genetic Metabolism, Xinhua Hospital, Shanghai Jiao Tong University School of Medicine, Shanghai, China; 3 Shanghai Institute for Pediatric Research, Xinhua Hospital, Shanghai Jiao Tong University School of Medicine, Shanghai, China

**Keywords:** Glutathione synthetase, 5-oxoprolinuria, Newborn, Metabolism, Mutation

## Abstract

Glutathione synthetase deficiency (GSSD) is a rare inborn error of glutathione metabolism with autosomal recessive inheritance. The severe form of the disease is characterized by acute metabolic acidosis, usually present in the neonatal period with hemolytic anemia and progressive encephalopathy. A case of a male newborn infant who had severe metabolic acidosis with high anion gap, hemolytic anemia, and hyperbilirubinemia is reported. A high level of 5-oxoproline was detected in his urine and a diagnosis of generalized GSSD was made. DNA sequence analysis revealed the infant to be compound heterozygous with two mutations, c.738dupG in exon 8 of *GSS* gene resulting in p.S247fs and a repetitive sequence in exon 3 of *GSS* gene. Treatment after diagnosis of GSSD included supplementation with antioxidants and oral sodium hydrogen bicarbonate. However, he maintained a variable degree of metabolic acidosis and succumbed shortly after his parents requested discontinuation of therapy because of dismal prognosis and medical futility when he was 18 days old.

## Introduction

Glutathione synthetase deficiency (GSSD) is a rare inborn error of glutathione metabolism with an autosomal recessive inheritance, which is caused by mutations in the *GSS* gene (OMIM 601002) (1). Patients may present with hemolytic anemia alone or together with acidosis and central nervous system impairment. Prior to 2007, only 70 patients were reported in more than 50 families worldwide (2). Due to increased awareness, several cases were reported in recent years, totaling more than 80 patients worldwide (3–8). Here, we report the case of a newborn infant with generalized GSSD. We cloned and characterized the human *GSS* gene and examined the DNA sequences of the patient with those of his parents. We identified two novel mutations at the GSS locus, which were inherited from either parent.

## Case report

The baby was admitted to our newborn intensive care unit for evaluation of intractable metabolic disease when he was 6 days old. He was born following an uncomplicated term pregnancy to a healthy mother. His parents were not consanguineous. Birth weight was 3100 g. His parents had one healthy 4-year-old daughter. Physical examination was normal at birth. The infant was breast fed. On the second day of life, he developed jaundice and tachypnea. A severe metabolic acidosis was detected on arterial blood gas for which he received intravenous sodium bicarbonate. The condition did not improve and he was referred to our hospital. On admission, his vital signs were as follows: temperature: 37.4°C; heart rate: 160/min; respiratory rate: 34/min; blood pressure: 78/39 mmHg. He was lethargic. Otherwise, the remainder of the physical examination was unremarkable.

A laboratory investigation revealed that the patient had severe metabolic acidosis without lactic acidosis or ketoacidosis. Blood ammonia was 106.0 μmol/L, liver and renal function tests were normal. He had a hemolytic anemia with progressive indirect hyperbilirubinemia. His hemoglobin was 53 g/L. Glucose-6-phosphate dehydrogenase (G6PD) activity was within normal limits. Blood metabolic screening and plasma amino acid chromatography showed that glutamic acid was increased. The urine gas chromatography/mass spectrometry showed massive excretion of 5-oxoproline. Table 1 shows laboratory findings of the patient on Day of Life #6.

**Table 1.** Laboratory findings on Day of Life #6.

Laboratory testing	Results
Complete blood count	Hb: 53 g/L, Hct: 17.6%, Reticulocyte count: 2.0%, WBC: 33,800/mm^3^, platelets: 157,000/mm^3^
Blood gases	pH: 7.00, pCO2: 25 mmHg, pO2: 71 mmHg, base excess: -23.1 mmol/L, HCO3-: 6.2 mmol/L
Anionic gap	20.8 mmol/L
Lactic acid	2.2 mmol/L
Blood ammonia	106.0 μmol/L
Total/direct bilirubin	438.5/71.7 μmol/L
Blood Glutamic acid	790.37 (100-400) μM
Urine 5-oxoproline	8723.54 (0-7.6) mmol/mol Cr
Appearance of erythrocytes on peripheral smear	Normal
Coomb's test	Negative
G6PD activity	3.80 (2.80-7.30) U·gHb^-1^·min^-1^
Pyruvate kinase activity	37.8 (15.2-53.3) U·gHb^-1^·min^-1^.

Hb: hemoglobin; Hct: hematocrit; WBC: white blood cells.

The second day after admission (Day of Life #7), the infant developed dyspnea, cyanosis and required mechanical ventilation. He underwent blood exchange because of hyperbilirubinemia and packed red blood cell transfusions. He had several seizure episodes. He was treated with intravenous sodium bicarbonate to mitigate his metabolic acidosis and he was weaned from mechanical ventilation. Following diagnosis of GSSD, supplementation of antioxidants (100 mg·kg^-1^·d^-1^ vitamin C and 10 mg·kg^-1^·d^-1^ vitamin E) was started. Nevertheless, he maintained a variable degree of moderate metabolic acidosis.

**Figure 1.**
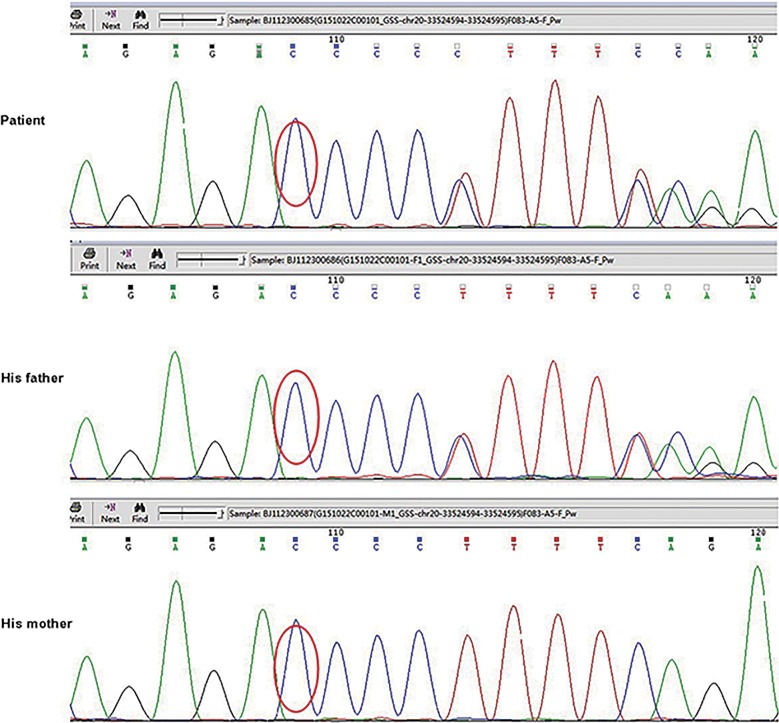
DNA sequence analysis of the patient and his parents. Nucleotide sequence showed heterozygous c.738dupG mutation in exon 8 of *GSS* gene in the patient and his father.

Informed consent was obtained from the patient’s parents. Genomic DNA was extracted from peripheral blood of the patient using the TIANamp Blood DNA Kit (Tiangen Biotech Co. Ltd., China) according to the manufacturer’s protocol. “Next-generation” DNA sequencing was performed on genomic DNA of the patient. The parental DNA samples were also analyzed to explore the mutation origin. The sequences were compared with the *GSS* (NM_000178) sequences in GeneBank. The novelty of mutation was ascertained with the database (http://www.hgmd.org/). Sequencing of the *GSS* gene revealed one previously unreported mutation. There was a heterozygous duplication of G at nucleotide 738 of the exon 8, which results in a frame shift from serine at 247. The mutation of c.738dupG; p.S247fs was also detected in his asymptomatic father’s DNA sample (Figure 1).

**Figure 2.**
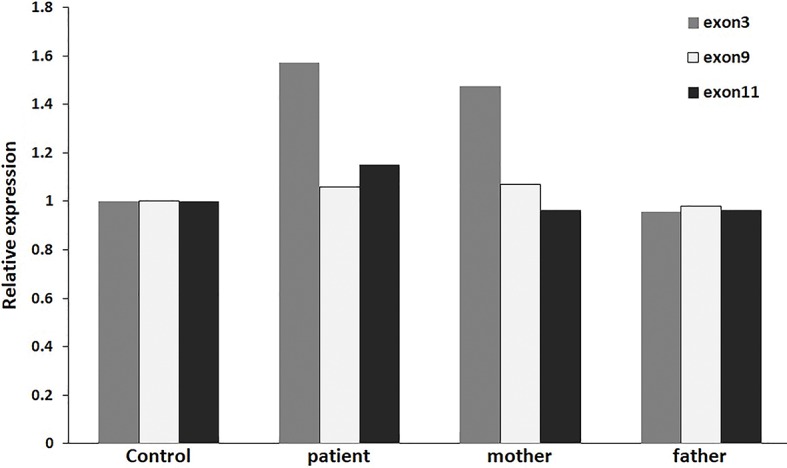
Relative expression of exon3, exon9 and exon11 of *GSS* gene in the patient and his parents. A relative expression of 1 indicates normality and a relative expression of about 1.5 indicates a repetitive sequence. qRT-PCR results showed that there was a repetitive sequence in exon3 of *GSS* gene in the patient and his mother (chr20:33530733-33530809).

“Next-generation” DNA sequencing also showed a possible repetitive sequence in exon 3 of *GSS* gene (Supplementary Figure S1). SYBR Real time polymerase chain reaction (qRT-PCR) was used to verify the possible repetitive sequence on the exons. The primers used were as follows: Exon 3F TGGAGTAGGGCTGTGAAT, Exon 3R AATGACTGAGGATGGGACT; Exon 9F AATTGGGAAGCACGTCTAC, Exon 9R TGCTGCACCTTCTTAGTC; Exon 11F CCTATATGGGGAGGAAATG, Exon 11R CTCAGGTTCGATCTTCTC; CPNE1-F CCTGAGTTCTCCAAGACT, CPNE1-R GTCATAGATTCCAAAGCGTAG. qRT-PCR results confirmed that the repetitive sequence in exon 3 of *GSS* gene (chr20:33530733-33530809) was inherited from his mother (Figure 2). These two novel heterozygous mutations were inherited from either parents and had not been reported previously.

On Day of Life #18, his parents requested discontinuation of therapy because of the grim prognosis and treatment futility. He died shortly after he was discharged from hospital. Parents declined request for autopsy.

## Ethics approval and consent to participate

All experiments complied with the requirement of the Ethics of Clinical Research issued by Ministry of Healthy, China. The research was approved by Xinhua Hospital, Shanghai Jiao Tong University School of Medicine, China.

## Discussion

Glutathione is a tripeptide containing glutamic acid, cysteine and glycine (1). Glutathione is present in millimolar concentrations in most mammalian cells and it is involved in several fundamental biological functions (9). Glutathione normally regulates its own biosynthesis by inhibiting γ-glutamylcysteine synthetase, the enzyme catalyzing the first step in the γ-glutamyl cycle. When glutathione concentration is reduced, γ-glutamylcysteine formation increases and this dipeptide is converted to 5-oxoproline in the plasma; some of the 5-oxoproline is excreted in urine. As it is a highly acidic compound, it causes metabolic acidosis (1).

GSSD is inherited as autosomal recessive trait. According to their clinical phenotype, patients with GSSD can be divided into three groups: mild, moderate, and severe. Mildly affected patients have mutations affecting the stability of the enzyme, causing a compensated hemolytic anemia. Moderately affected patients have hemolytic anemia and metabolic acidosis, resulting in low levels of glutathione in erythrocytes. Severely affected patients also develop severe metabolic acidosis, elevated levels of 5-oxoproline in urine and blood, increased susceptibility to bacterial infections, and central nervous system disorders showing mental retardation, seizures, spasticity, ataxia and intention tremors, pathological electroretinograms and retinal pigmentation (10). Clinical signs usually first appear during the neonatal period. After the neonatal period, the condition usually stabilizes but may exacerbate during an infection due to severe acidosis or electrolyte imbalance (11).

Diagnosis is usually made clinically in addition to massive excretion of L-5-oxoproline (up to 1 g·kg^-1^·day^-1^) in the urine. Decreased activity of GSS can be demonstrated in erythrocytes, leukocytes and cultured fibroblasts. 5-oxoprolinuria is caused by GSSD or 5-oxoprolinase deficiency. 5-oxoprolinase deficient patients have normal acid-base status and do not have hemolytic anemia. Transient 5-oxoprolinuria (pyroglutamic aciduria) with systemic acidosis has been reported in an adult receiving antibiotic therapy (12). 5-oxoprolinuria can also be caused by acetaminophen overuse, vigabatrin or flucloxacillin (13–15). However, the levels of pyroglutamate are much less marked in these conditions than in the inherited disorders (15). The present case had severe metabolic acidosis with a high anionic gap, severe hemolytic anemia, hyperbilirubinemia, and high levels of 5-oxoprolinuria. GSS activity was not assessed in erythrocytes because of lack of appropriate lab in our hospital.

Treatment involves correction of metabolic acidosis initially by parenteral compounds followed by oral maintenance therapy, antibiotic treatment if there is an infection, and supportive care. In the neonatal period, it is especially important to prevent hyperbilirubinemia in order to protect the brain from kernicterus. Anemia often needs to be treated with blood transfusion. Blood filtration is an option in more severe cases. In our case, hyperbilirubinemia and anemia was alleviated after blood exchange. As there is increased sensitivity to oxidative stress, such anti-oxidative agents as vitamin E, C, and N-acetylcysteine have been used (16). Drugs avoided in G6PD deficiency should also be restricted in GSSD. Oral administration of glutathione analogues have been shown to increase glutathione concentration in leukocytes and plasma with no effect on 5-oxoproline excretion in urine (16). High doses of α-tocopherol may improve erythrocyte survival in GSS deficient patients. Recently, it was reported that sodium bicarbonate was effective for treatment of chronic metabolic acidosis in GS deficiency (8). Prognosis depends on the type of mutation, severity of acidosis, associated bacterial infections and the quality of supportive therapy. A long-term follow up study of 28 patients with GSSD has indicated that the factors most predictive of survival and long-term outcome are early diagnosis and early supplementation with vitamins C and E (11). Prenatal diagnosis of GSSD is possible by analyzing 5-oxoproline in amniotic fluid, or by enzyme analysis in cultured amniocytes or chorionic villi samples (2).

Human GSS is a homodimer of two 52 kDa subunits that catalyzes the synthesis of glutathione by adding glycine to the dipeptide. GSS gene is located on chromosome 20q11.2 and includes 13 exons (1). Several studies showed that many disease-causing mutations at the GSS locus were identified (17–19). A considerable fraction of patients with inherited GSSD exhibit splice mutations that are not readily detected by PCR-mediated sequence analysis of individual exons and exon-intron boundaries from genomic DNA, and those patients who carry mutations leading to abnormal splicing patterns can be diagnosed using RT-PCR (19).

To date, 32 different mutations from more than 70 GSS deficient patients have been reported (http://www.hgmd.cf.ac.uk/). In this study, two novel mutations on GSS gene were found. Due to the triplet nature of gene expression by codons, the insertion or the repetitive sequence in exon can alter the reading frame, resulting in a completely different translation product from the original. The parents harbored each mutation of the patient. The findings meet Mendel’s law of inheritance. Li et al. (6) reported three Chinese patients with GSSD. In that study, c.491G>A mutation in GSS gene accounted for 66.67% of the alleles of 3 cases (6). However, the case in this study had different mutation of GSS gene. In certain states in the USA, pyroglutamic aciduria is included in the neonatal screening programs (4).

## Supplementary material

Click here to view [pdf].

## References

[1] Al-Jishi E, Meyer BF, Rashed MS, Al-Essa M, Al-Hamed MH, Sakati N (1999). Clinical, biochemical, and molecular characterization of patients with glutathione synthetase deficiency. Clin Genet.

[2] Larsson A, Ristoff E, Anderson ME, Scriver CR, Beaudet AL, Sly WS, Valle D, Vogelstein B, Childs B (2005). Glutathione synthetase deficiency and other disorders of the gamma-glutamyl cycle. The metabolic and molecular bases of inherited disease.

[3] Sykut-Cegielska J, Jurecka A, Taybert J, Gradowska W, Pajdowska M, Pronicka E (2005). Trial of erythropoietin treatment in a boy with glutathione synthetase deficiency. J Inherit Metab Dis.

[B04] Simon E, Vogel M, Fingerhut R, Ristoff E, Mayatepek E, Spiekerkötter U (2009). Diagnosis of glutathione synthetase deficiency in newborn screening. J Inherit Metab Dis.

[B05] Ben Ameur S, Aloulou H, Nasrallah F, Kamoun T, Kaabachi N, Hachicha M (2015). Hemolytic anemia and metabolic acidosis: think about glutathione synthetase deficiency. Fetal Pediatr Pathol.

[B06] Li X, Ding Y, Liu Y, Ma Y, Song J, Wang Q (2015). Five Chinese patients with 5-oxoprolinuria due to glutathione synthetase and 5-oxoprolinase deficiencies. Brain Dev.

[B07] Şekeroğlu HT, Hismi B, Kadayifcilar S, Coskun T (2015). Fundus autofluorescence and optical coherence tomography findings in glutathione synthetase deficiency. J AAPOS.

[8] Gündüz M, Ünal Ö, Kavurt S, Türk E, Mungan NÖ (2016). Clinical findings and effect of sodium hydrogen carbonate in patients with glutathione synthetase deficiency. J Pediatr Endocrinol Metab.

[9] Ristoff E, Larsson A (2007). Inborn errors in the metabolism of glutathione. Orphanet J Rare Dis.

[10] Njalsson R (2005). Glutathione synthetase deficiency. Cell Mol Life Sci.

[11] Ristoff E, Mayatepek E, Larsson A (2001). Long-term clinical outcome in patients with glutathione synthetase deficiency. J Pediatr.

[12] Croal BL, Glen AC, Kelly CJ, Logan RW (1998). Transient 5-oxoprolinuria (pyroglutamic aciduria) with systemic acidosis in an adult receiving antibiotic therapy. Clin Chem.

[13] Armenian P, Gerona RR, Blanc PD, Wu AH, Mookherjee S (2012). 5-oxoprolinemia causing elevated anion gap metabolic acidosis in the setting of acetaminophen use. J Emerg Med.

[B14] Verma R, Polsani KR, Wilt J, Loehrke ME (2012). 5-Oxoprolinuria as a cause of high anion gap metabolic acidosis. Br J Clin Pharmacol.

[15] Milosevic S, Tran K, O’Brien B (2013). A rare cause of high anion gap metabolic acidosis. Intern Med J.

[16] Jain A, Buist NR, Kennaway NG, Powell BR, Auld PA, Martensson J (1994). Effects of ascorbate or N-acetylcysteine treatment in a patient with hereditary glutathione synthetase deficiency. J Pediatr.

[17] Shi ZZ, Habib GM, Rhead WJ, Gahl WA, He X, Sazer S (1996). Mutations in the glutathione synthetase gene cause 5-oxoprolinuria. Nat Genet.

[B18] Dahl N, Pigg M, Ristoff E, Gali R, Carlsson B, Mannervik B (1997). Missense mutations in the human glutathione synthetase gene result in severe metabolic acidosis, 5-oxoprolinuria, hemolytic anemia and neurological dysfunction. Hum Mol Genet.

[19] Njålsson R, Carlsson K, Winkler A, Larsson A, Norgren S (2003). Diagnostics in patients with glutathione synthetase deficiency but without mutations in the exons of the GSS gene. Hum Mutat.

